# Prognostic role of platelet to lymphocyte ratio in pancreatic cancers: A meta-analysis including 3028 patients

**DOI:** 10.1097/MD.0000000000009616

**Published:** 2018-02-23

**Authors:** Wendi Li, Lianyuan Tao, Meng Lu, Dianrong Xiu

**Affiliations:** Department of General Surgery, Peking University Third Hospital, Beijing, China.

**Keywords:** meta-analysis, pancreatic cancer, platelet to lymphocyte ratio, prognosis

## Abstract

**Background:**

Platelet to lymphocyte ratio (PLR) was recently reported being associated with the prognosis of pancreatic cancer (PC), but the prognostic value of PLR in pancreatic cancer remains inconsistent. We conduct a meta-analysis to evaluate the prognostic role of PLR in patients with PC.

**Methods:**

PubMed, Embase, Cochrane Library, and Web of Science were systematically searched for eligible studies which investigated the relationship between PLR and clinical outcome of patients with pancreatic cancer. Pooled hazard ratios (HRs) and 95% confidence intervals (CIs) were calculated to evaluate the prognostic role of PLR in overall survival (OS) and progression-free survival (PFS)/time to progression (TTP).

**Results:**

A total of 16 studies comprising 3028 patients with PC were enrolled in this meta-analysis. Pooled analysis demonstrated that elevated PLR predicted a poor OS (HR = 1.22, 95% CI: 1.09–1.36, *P* < .001). Prognostic role of PLR on OS were significant in subgroup of Asians (HR = 1.22, 95% CI: 1.11–1.34, *P* < .001), patients treated with chemotherapy (HR = 1.18, 95% CI: 1.04–1.35, *P* = .01) and mixed methods (HR = 1.29, 95% CI: 1.07–1.57, *P* = .009), American joint committee on cancer (AJCC) stage of III–IV (HR = 1.22, 95% CI: 1.09–1.36, *P* < .001), pathological subtype of pancreatic adenocarcinoma (HR = 1.21, 95% CI: 1.08–1.36, *P* = .001), and cut-off value of PLR ≥160 (HR = 1.48, 95% CI: 1.25–1.75, *P* < .001).

**Conclusions:**

An elevated PLR is associated with unfavorable overall survival in patients with pancreatic cancer.

## 1 Introduction

Pancreatic cancer (PC) remains an extremely aggressive malignancy, as it is the fourth leading cancer-related death worldwide.^[1]^ Despite progress has been made in the diagnosis and therapies of PC, the prognosis of which is still poor. Besides tumor-node-metastasis (TNM) staging system, effective and convenient prognostic biomarkers are needed. Therefore, it is of paramount importance for us to identify a better prognostic biomarker for prognosis of PC, which could facilitate the selection of individual therapeutic strategy in clinical management.

Recently, systemic inflammation status has been revealed to be involved in tumor development and progression.^[2–4]^ Serum biomarkers of inflammation can be readily obtained through peripheral blood samples, such as C-reactive protein, neutrophil to lymphocyte ratio (NLR), lymphocyte to monocyte ratio (LMR), and platelet to lymphocyte ratio (PLR). Such biomarkers have been revealed to have prognostic value for a variety of malignancies, including colorectal cancer,^[5]^ lung cancer,^[6]^ ovarian cancer,^[7]^ and so on. A multitude of studies indicated that an elevated NLR predicted poor survival in patients with PC.^[8–12]^ Evidence has suggested that LMR was associated with favorable survival in PC patients.^[13–16]^ Nevertheless, the prognostic value of PLR for PC patients is still controversial and has not yet been systematic analyzed. Therefore, we conducted this meta-analysis to elucidate the prognostic value of PLR on OS and progression-free survival (PFS)/time to progression (TTP) in PC patients. As far as we know, this is the first meta-analysis explored the connection between PLR and prognosis in PC.

## 2 Materials and methods

### 2.1 Search strategies

This study was conducted according to the Preferred Reporting Items for Systematic Reviews and Meta-Analyses (PRISMA) statement.^[17]^ We performed a comprehensive literature search of PubMed, Embase, Cochrane Library, and Web of Science up to June 30, 2017. The following terms were used: “PLR” (e.g., “platelet lymphocyte ratio,” “platelet–lymphocyte ratio,” “platelet to lymphocyte ratio,” and “platelet-to-lymphocyte ratio”) and “PC” (e.g., “pancreatic cancer,” “pancreatic carcinoma,” “pancreatic tumor,” “pancreatic neoplasms,” “pancreatic adenocarcinoma,” and “PDAC,”). References of relevant studies were also checked for eligible studies. This study was approved by the Clinical Ethics Committee of Peking University Third Hospital.

### 2.2 Inclusion and exclusion criteria

Inclusion criteria for selecting the studies were as follows: patients with PC were pathological examination confirmed; studies described the correlation of PLR with overall survival (OS) and/or cancer-specific survival (CSS) and/or time to progression (TTP) and/or disease-free survival (DFS) and/or progression-free survival (PFS); hazard ratios (HRs) with 95% confidence intervals (95% CIs) were reported. Exclusion criteria were: letters, case reports, abstracts, editorials, comments, and reviews; studies with duplicate data and repeat analyses; studies were not written in English.

### 2.3 Data extraction

The following information was captured by 2 independent authors (WL and LT): first author's name, year of publication, country, ethnicity, survival analysis methods (multivariate/univariate), time of follow-up and survival outcome (OS, CSS, PFS, and TTP). Age of patients, sample size (male and female), pathological subtype, American joint committee on cancer (AJCC) stage, treatment strategy, cut-off values of PLR, and consideration of receiver operating characteristic (ROC) curves for selection of cut-off. HRs as well as their 95%CIs for OS, CSS, PFS, and TTP. HRs and their 95%CIs were extracted from multivariable analyses in consideration of confounding factors. If multivariable analyses were not available, HRs from univariable analyses were extracted. Any discrepancies were resolved by discussion to reach a consensus.

### 2.4 Qualitative assessment

The quality assessment of each study was independently performed by 2 authors (WL and ML) according to the Newcastle-Ottawa Scale (NOS),^[18]^ which included criteria of sample selection (0–4 points), comparability (0–2 points), and outcome assessment (0–3 points). Studies with NOS score of ≥6 were assigned as high-quality studies.

### 2.5 Statistical analysis

STATA version 13.0 (StataCorp, CollegeStation, TX) was used for statistical analysis. The chi-square-based *Q*-statistic test and the *I*-squared statistic were performed to assess the inter-study heterogeneity.^[19]^ A fixed-effects model was used if there was no significant heterogeneity (Ph >0.10 for the *Q*-test and *I*^2^ <50%). Otherwise, a random-effects model was selected. The aggregated HRs and 95% CIs were applied to access the prognostic value of PLR on OS and PFS/TTP. Subgroup analyses were conducted on the following items to explore the heterogeneity among the results of different studies: ethnicity, treatment, AJCC stage, sample size, HR analysis method, pathological type, and cut-off for PLR. Sensitivity analyses were conducted by evaluating result stability after sequential omission of each study. Egger linear regression test was used to estimate publication bias.^[20]^ A trim and fill method was applied to estimate asymmetry in the funnel plot.^[21]^ All statistical tests were 2-tailed, and *P* < .05 was defined as statistical significance.

## 3 Results

### 3.1 Search results and study characteristics

**Figure 1:**
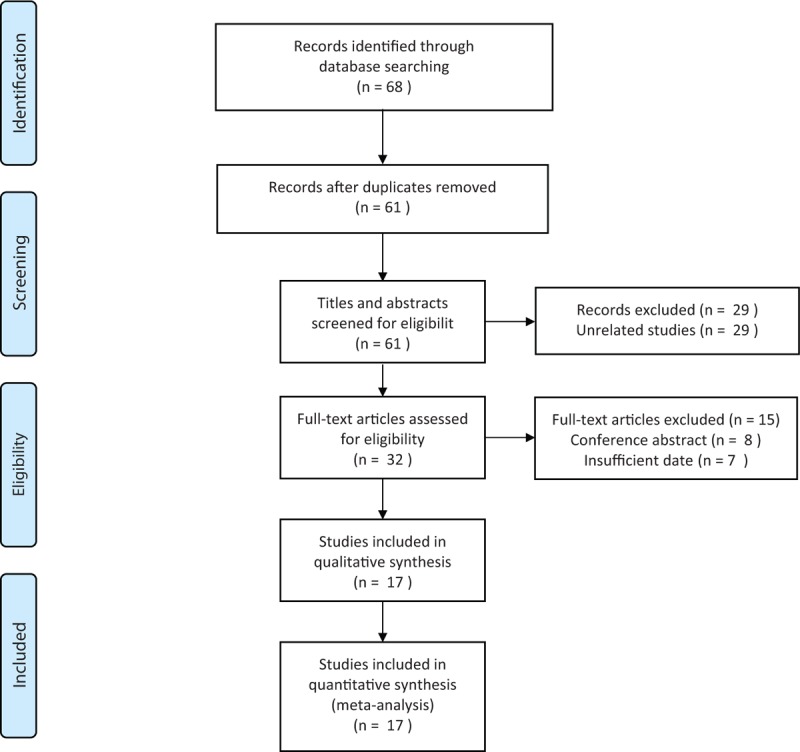
Flow diagram of the study selection in the analysis.

The initial search algorithm retrieved a total of 68 studies, of which 7 were duplicates. After titles and abstracts assessed for eligibility, the left 32 articles were enrolled for full-text articles screened. Of them, 15 articles were excluded due to conference abstract or insufficient data. Finally, we selected 17 studies^[9,22–37]^ for the meta-analysis following the Preferred Reporting Items for Systematic Reviews and Meta-Analyses (PRISMA) statement.^[17]^ The processes of study selection were shown in the PRISMA flow diagram (Fig. 1).

Seventeen studies comprising 3028 patients were included for the present analysis and characteristics are summarized in Table 1. Qi et al^[22,23]^ reported 2 independent studies, and samples came from the same institution in China, but the enrolled patients were non-repetitive. Stotz et al^[37]^ reported 2 groups of pancreatic cancer patients, in which the relationship between the PLR and CSS was available. These studies were published between 2010 and 2017 and contained sample sizes ranged from 37 to 440. There were 13 studies evaluated Asian patients, and the other 4 evaluated Caucasian patients. “Caucasian” means Caucasian race and the majority of the patients enrolled in non-Asian studies were Caucasian. The treatments were surgery, chemotherapy, radiotherapy, chemoradiotherapy, and mixed methods. There were 16 studies reported the association between PLR and OS, and 2 studies investigated prognostic value of PLR for PFS/TTP. Cutoff values of PLR ranged from 126 to 300. The Newcastle-Ottawa scale (NOS) scores of the studies varied from 6 to 8.^[18]^

**Table 1:**
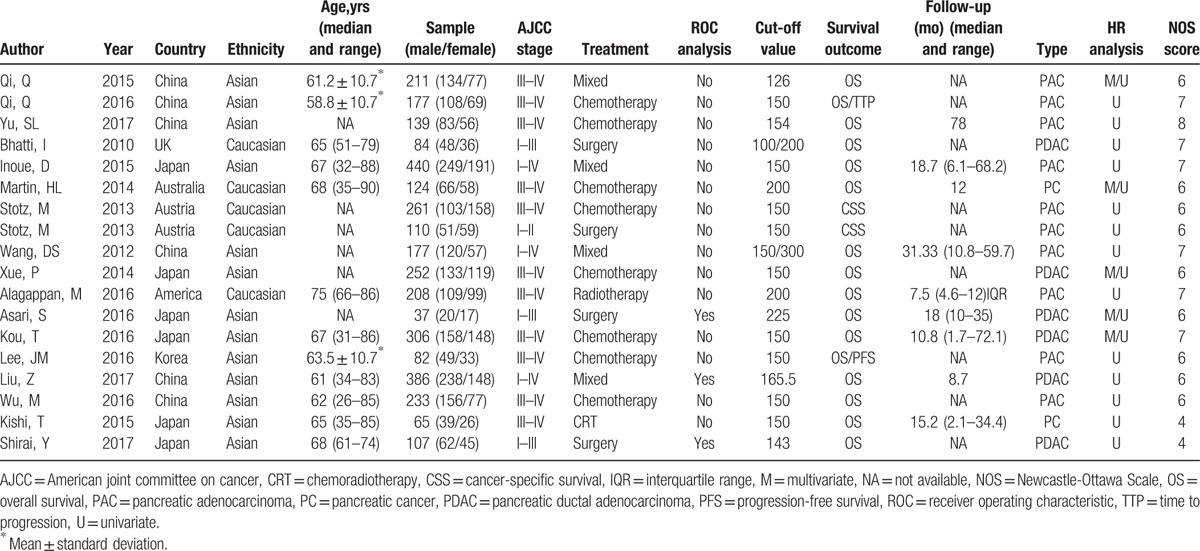
Main characteristics of all the studies included in the meta-analysis.

### 3.2 Meta-analysis

#### 3.2.1 The prognostic value of PLR in OS

**Figure 2:**
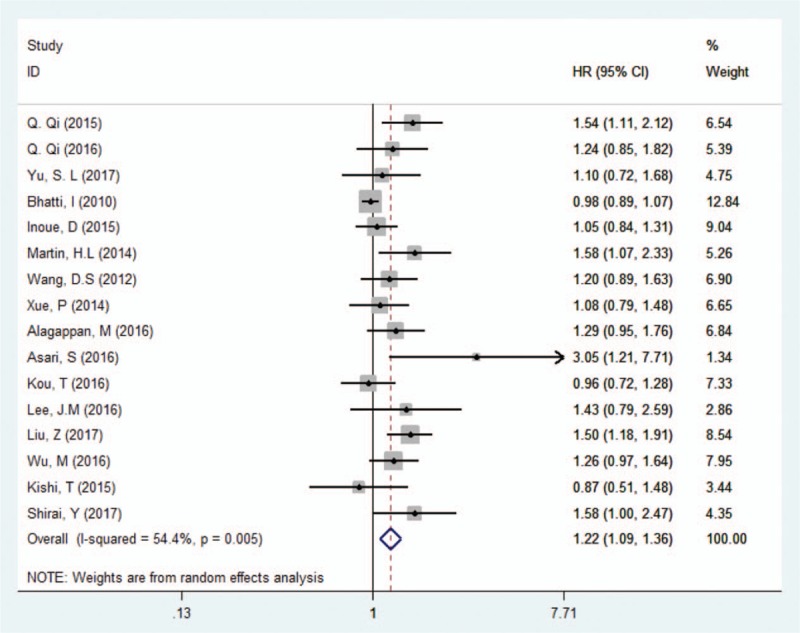
Forest plots for the association between PLR and OS in PC. OS = overall survival, PC = pancreatic cancer, PLR = platelet–lymphocyte ratio.

Sixteen studies comprising 3028 patients reported the association between PLR and OS. The pooled analysis suggested that an elevated PLR predicted an unfavorable OS (HR = 1.22, 95% CI: 1.09–1.36, *P* < .001). There was heterogeneity of included studies and a random-effects model was used (Ph = 0.005; *I*^2^ = 54%) (Fig. 2).

#### 3.2.2 The prognostic value of PLR in PFS/TTP

**Figure 3:**
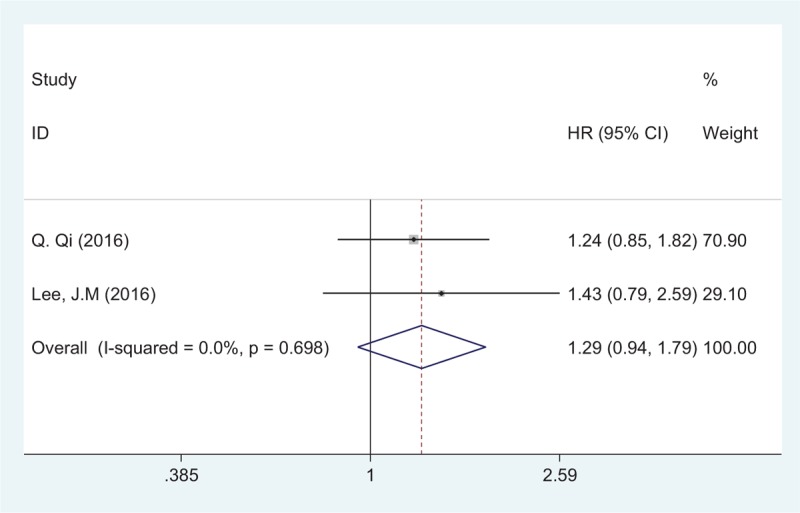
Forest plots for the association between PLR and PFS/TTP. PFS/TTP = progression-free survival/time to progression, PLR = platelet–lymphocyte ratio.

Two studies comprising 259 patients evaluated the relationship between PLR and PFS/TTP. The fixed-effects model was used for analysis because there was no significant heterogeneity (Ph = 0.698; *I*^2^ = 0%). Pooled data indicated that PLR has no prognostic value for PFS/TTP in patients with advanced pancreatic adenocarcinoma (HR = 1.29, 95% CI: 0.94–1.79, *P* = .24) (Fig. 3).

#### 3.2.3 Subgroup analysis

We conducted subgroup analyses for PLR and OS based on ethnicity, treatment, AJCC stage, sample size, HR analysis method, pathological type, and cut-off for PLR (Table 2). Subgroup analysis according to ethnicity showed that PLR had a prognostic role for OS in Asians (HR = 1.22, 95% CI: 1.11–1.34, *P* < .001). In the subgroup of the treatment, increased PLR predicted a pool OS in patients treated with chemotherapy (HR = 1.18, 95% CI: 1.04–1.35, *P* = .01) and mixed methods (HR = 1.29, 95% CI: 1.07–1.57, *P* = .009). When stratified by AJCC stage, the analyses indicated that PLR was a prognostic marker in patients with AJCC stage of III–IV (HR = 1.22, 95% CI: 1.09–1.36, *P* < .001). Subgroup analysis for pathological type indicated that PLR had prognostic value for pancreatic adenocarcinoma (HR = 1.21, 95% CI: 1.08–1.36, *P* = .001). In the subgroup of the cut-off value for PLR, the results indicated that increased PLR was significantly associated with unfavorable OS in studies of cut-off value ≥160 (HR = 1.48, 95% CI: 1.25–1.75, *P* < .001). PLR showed prognostic value regardless of sample size and HR analysis method. Details are shown in Table 2.

**Table 2:**
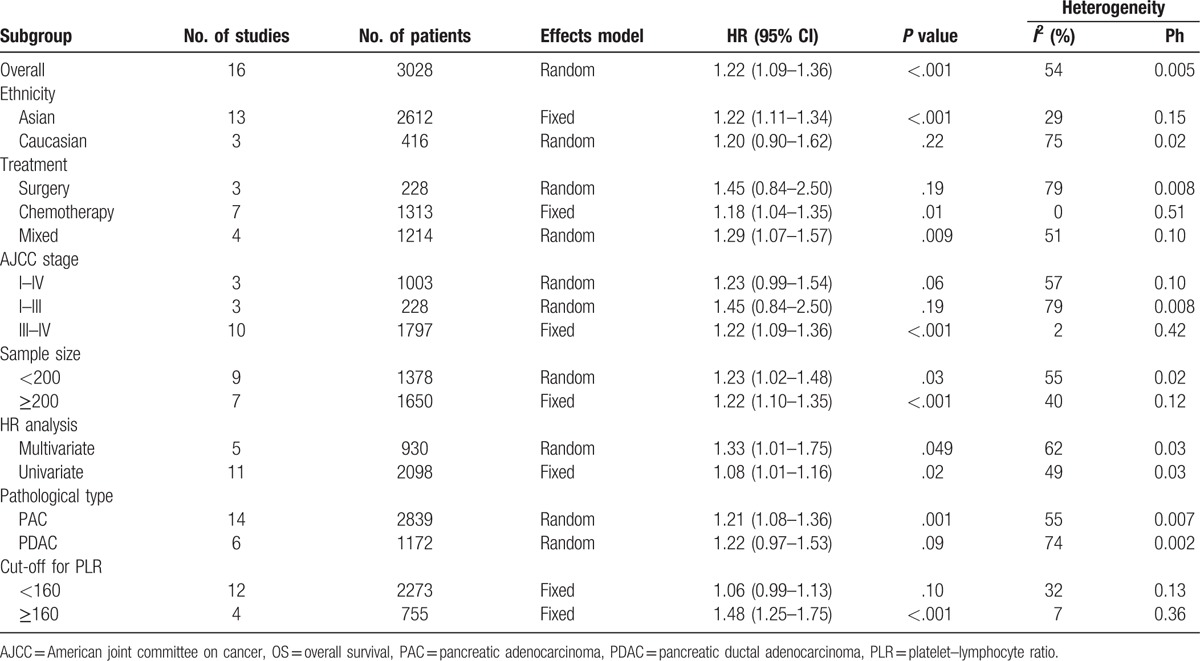
Subgroup analyses for the association between PLR and OS.

### 3.3 Sensitivity analysis

**Figure 4:**
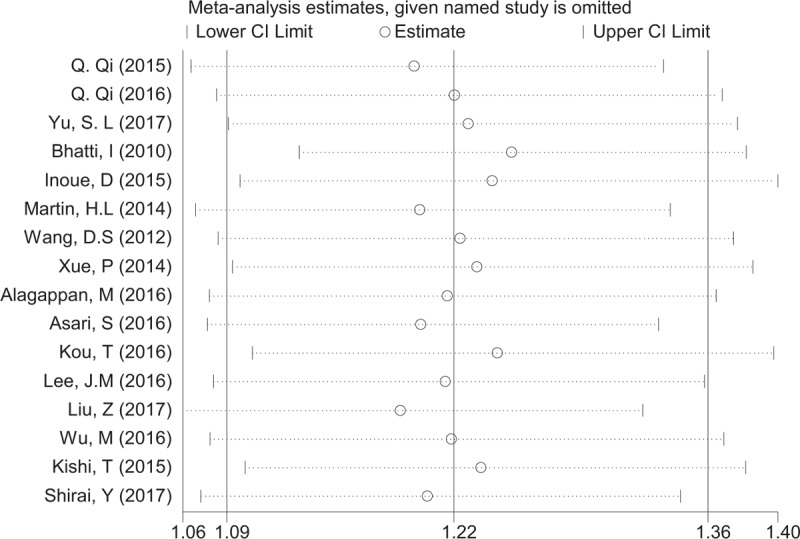
Sensitivity analyses for confirming robustness of OS by removing 1 study each time. OS = overall survival.

Sensitivity analysis was performed to assess the stability of the results. Each single study was removed to check the influence of individual data sets on the pooled HR of OS. The result showed that the combined HR was not obviously influenced by any single study, which indicated the robustness of the outcome of the study (Fig. 4).

### 3.4 Publication bias

**Figure 5:**
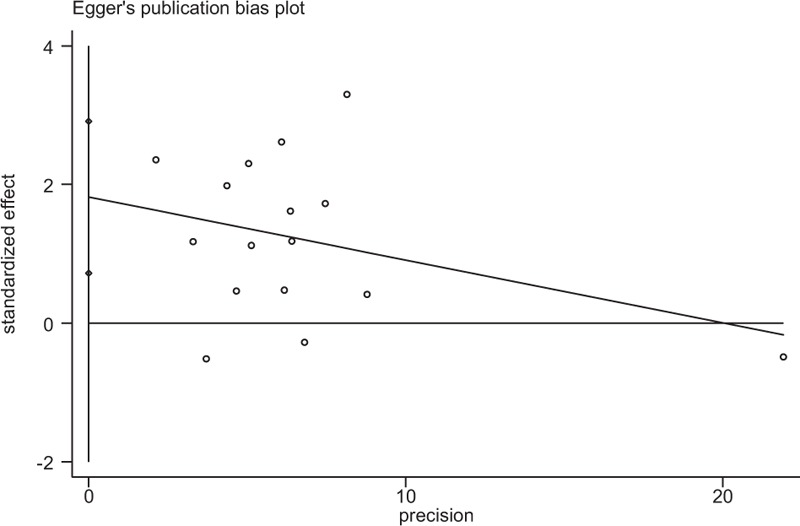
Egger publication bias plot for studies investigated OS in PC. (*P* = .003). OS = overall survival; PC = pancreatic cancer.

**Figure 6:**
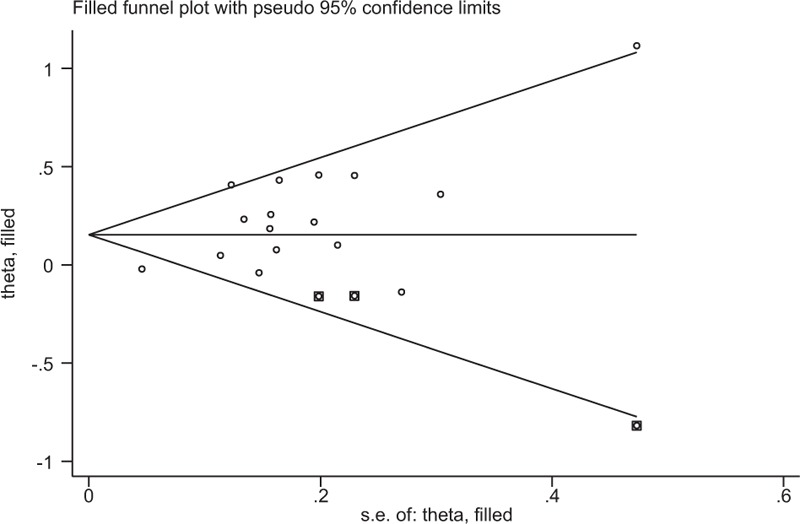
Funnel plot adjusted using a trim and fill method for studies investigated OS in PC. Diamonds: included studies; diamonds in squares: presumed missing studies. OS = overall survival; PC = pancreatic cancer.

Egger publication bias plot test was used to estimate the publication bias (Fig. 5). There was publication bias for studies investigated OS in PC (*P* = .003). Therefore, a trim and fill method was used to fill unpublished studies, and the recalculated combined HR of OS did not be significantly affected by filling 3 unpublished studies (HR = 1.16; 95% CI, 1.04–1.30; *P* = .006; Fig. 6).

## 4 Discussion

PLR was being confirmed as a significant prognostic marker in colorectal cancer (CRC) and non-small cell lung cancer(s) (NSCLC). Tan et al^[38]^ conducted a systematic review and meta-analysis to prove that peripheral blood PLR can be used as a predictor of OS in patients with CRC. Zhao et al^[39]^ also performed a meta-analysis suggested that elevated PLR might be a predicative factor of poor prognosis for NSCLC patients.

Our meta-analysis combined 16 studies involving 3028 patients and indicated that the elevated PLR significantly predicted unfavorable OS in patients with PC. Subgroup analyses suggested that increased PLR was associated with poor OS in Asian populations. Furthermore, subgroup analyses for treatments implied that higher prognostic value of PLR in OS was found in patients treated with chemotherapy and mixed methods. In addition, the stratified analyses showed that PLR have higher prognostic value for OS in PC patients with AJCC stage of III–IV. As for pathological subtype of PC, higher negative effect of elevated PLR on OS was observed in patients with pancreatic adenocarcinoma (PAC), and the prognostic value of PLR was impaired for pancreatic ductal adenocarcinoma (PDAC). The cut-off value of PLR for OS ranged from 126 to 300, and our stratified analyses suggested that PLR with cut-off value ≥160 may have more discriminative prognostic value for OS. Owing to the limited studies, the meta-analysis did not demonstrate prognostic value of PLR with PFS/TTP in PC.

The bilateral influence of systemic inflammatory response and tumor progression is involved in the prognosis of PC patients.^[40]^ The exact mechanisms by which PLR predicts OS of PC patients are still undefined. Platelets are recruited and adhere to tumor cells through platelet receptors, such as glycoprotein IIb/IIIa and P-selectin.^[41,42]^ Additionally, platelets secrete tumor growth factors, such as vascular endothelial growth factor (VEGF), platelet-derived growth factor (PDGF), transforming growth factor-β1 (TGFβ1), and insulin-like growth factor-1 (IGF1).^[43]^ These factors play a crucial role in tumorigenesis, angiogenesis, and metastasis. Furthermore, platelets help tumor cells escape from the immune system by surrounding circulating tumor cells (CTCs) through platelets aggregation.^[44]^

Lymphocytes are essential components of the immune system. Tumor-infiltrating lymphocytes (TILs) are vital components of the antitumor immune microenvironment, and are cellular basis of immunosurveillance against tumor cells.^[45]^ CD4^+^ and CD8^+^ T lymphocytes induce cytotoxic cell death and inhibit tumor cell proliferation and migration in antitumor immunitive reaction.^[46,47]^ Furthermore, lymphocytopenia has been clarified to be a prognostic factor of OS in PC.^[48]^ Therefore, the ratio of increased platelets and decreased lymphocytes has prognostic value for patients with PC. According to the present research, the association of platelet and lymphocyte in pancreatic cancers is that platelet help tumor cells escape from the immune system which based on lymphocyte by surrounding CTCs through platelets aggregation.

There were several limitations to this study. First, all enrolled studies were retrospective, which may exist some biases. Second, heterogeneity was found among studies for OS. Therefore, subgroup analyses were conducted to adjust for heterogeneity. Most of the studies used univariate analysis, so there was not enough evidence to prove that PLR was an independent prognostic factor for PC. Third, potential publication bias was observed in OS meta-analysis. However, through trim and fill analysis, the recalculated results confirmed the robustness of the prognostic role of PLR in OS. Finally, only English publications were enrolled, language bias cannot be excluded.

In conclusion, an elevated PLR is associated with unfavorable OS in patients with pancreatic cancer. The PLR could be a convenient and economical prognostic biomarker of pancreatic cancer in the clinical practice, which could facilitate the selection of individual therapeutic strategy for patients with PC.
